# Prevalence of Coronary Microvascular Dysfunction and Epicardial Spasm in Patients With Angina and Myocardial Bridge

**DOI:** 10.1016/j.jscai.2024.102196

**Published:** 2024-08-12

**Authors:** Tess E. Allan, Michael M. Mayer, Steven E.S. Miner, Hena Patel, Amit R. Patel, Husam H. Balkhy, Jonathan D. Paul, Atman P. Shah, Sandeep Nathan, John E.A. Blair

**Affiliations:** a Section of Cardiology, Department of Medicine, The University of Chicago Medical Center, Chicago, Illinois; b Division of Cardiology, Southlake Regional Health Centre, Newmarket, Ontario, Canada; c School of Kinesiology and Health Science, York University, Toronto, Ontario, Canada; d Department of Medicine, University of Toronto, Toronto, Ontario, Canada; e Division of Cardiovascular Medicine, Department of Medicine, University of Virginia, Charlottesville, Virginia; f Division of Cardiology, Department of Medicine, University of Washington, Seattle, Washington

**Keywords:** chest pain, coronary microvascular dysfunction, epicardial spasm, ischemia with nonobstructive coronary arteries, microvascular spasm, myocardial bridge

## Abstract

**Background:**

Myocardial bridges (MB) are prevalent but not universally associated with angina. The mechanisms linking MB and angina are poorly defined. The objective of this study was to determine the prevalence of epicardial spasm, microvascular spasm, and/or endothelium-independent coronary microvascular dysfunction (CMD) in patients with MB which might explain symptoms.

**Methods:**

Patients with known MB and chest pain at the University of Chicago Medical Center between 2020-2023 were included. All patients underwent dobutamine testing with measurement of resting full-cycle ratio to determine hemodynamic significance (resting full-cycle ratio ≤0.76). Endothelium-independent CMD was defined as coronary flow reserve <2.0 or index of microvascular resistance ≥25 on adenosine testing. Microvascular spasm was defined as chest pain and electrocardiogram changes with nonischemic fractional flow reserve with acetylcholine. Epicardial spasm was defined as dynamic stenosis of >90% of the epicardial vessel or ischemic fractional flow reserve (≤0.8) with acetylcholine.

**Results:**

A total of 30 patients (mean age, 47 ± 10 years; 60% female) with MB were studied. Endothelium-independent CMD, microvascular spasm, and epicardial spasm occurred commonly in 60%, 29%, and 37% of patients respectively, with 77% having at least one abnormality. The MB was hemodynamically significant in 47% of patients, and the prevalence of these coexisting conditions was not affected by hemodynamic significance.

**Conclusions:**

Epicardial spasm, microvascular spasm, and endothelium-independent CMD are prevalent in patients presenting with known MB and chest pain irrespective of the hemodynamic significance of the bridge. Invasive coronary function testing may play an important role in uncovering alternative explanations for angina in patients with known MB.

## Introduction

Myocardial bridges (MB) are common in the general population and can be identified using coronary angiography, cardiac computed tomographic angiography, or at autopsy. The overall prevalence of MB as described in several meta-analyses is estimated to be around 25%^1^ to 42%^2^ on autopsy with lower rates around 6% cited when angiography is used.^1^ Our group has demonstrated that prevalence of incidental MB found on post heart transplant assessment was 67.4% when intravascular ultrasound was used.^3^ The bridge segment is most commonly located in the left anterior descending artery (LAD)^1^^,^^2^ and causes external compression of the vessel during systole. This compression can lead to inducible ischemia with exercise, stress, or stimulation with dobutamine (Dob) infusion, and has been related to the degree of angiographic compression.^4^ The reduction in vessel diameter that occurs during systole can persist during diastole.^2^

Because MB is highly prevalent in the general population, they are also prevalent in patients presenting with angina. This can pose a diagnostic dilemma as many bridges are not hemodynamically (HD) significant and presence of an MB may not explain the chest pain syndrome. Available literature on rates of HD significant bridges is limited. In a study of patients presenting with chest pain and MB, 42% were found to have HD significant bridges as determined by invasive functional testing.^5^ A subsequent study of patients with chest pain and MB found that 32% of patients had evidence of myocardial ischemia on stress echocardiography.^6^ Given that all patients in these studies had chest pain, the reported prevalence of HD significant bridges is likely higher in this population than in a population without chest pain. Physiologic evaluation of MB aids in identification of HD significant bridge segments^7^ and can guide the necessity of medical and surgical therapy.

Given the prevalence of MB but relatively low rates of hemodynamic significance, it is possible that patients with angina and MB may also have concomitant functional abnormalities including endothelium-independent coronary microvascular dysfunction (CMD), microvascular spasm, and/or epicardial vasospasm contributing to or even completely explaining their angina. CMD is an important cause of chest pain and ischemia with no obstructive coronary artery disease^8^ with one study reporting a CMD prevalence of 66% in patients presenting with chest pain.^9^ MB has been associated with even higher rates of epicardial and microvascular spasm on acetylcholine (ACh) testing.^10^, ^11^, ^12^ Current studies have not evaluated the effect of invasively determined hemodynamic significance of the MB on the prevalence of other functional abnormalities. Additionally, no studies to date have assessed thoroughly for epicardial spasm, microvascular spasm, and endothelium-independent CMD using definitions recommended in recent consensus guidelines.^13^ The aim of this study is to describe the coronary endotypes of patients with known MB being evaluated for surgical therapy, stratified by the hemodynamic significance of the bridge.

## Methods

### Study population and clinical parameters

This was a prospective study of 30 patients presenting with chest pain initially attributed to an MB referred to the University of Chicago Medical Center for further evaluation between January 2020 and June 2023. We perform robotic surgical unroofing of MB patients at our center,^14^ so these evaluations are designed to determine candidacy for this surgery. We excluded patients with significant coronary artery disease (stenosis >50% or fractional flow reserve [FFR] ≤0.8), cardiac transplantation, or more than mild valvular dysfunction. Baseline clinical information is recorded at the time of the precatheterization visit and was abstracted from the electronic medical record.

### Coronary function testing

Prior to cardiac catheterization, caffeine, nitrates, and calcium channel blockers were held for 48 hours. The radial approach was utilized in all patients and a modified radial cocktail of heparin and nitroglycerin (no calcium channel blocker) was given. Diagnostic angiography was performed according to standard procedures outlined in the 2021 SCAI expert consensus update on best practices in the cardiac catheterization laboratory.^15^ For the function testing, a guide catheter was placed in the left main coronary artery, and heparin was administered for a target-activated clotting time of 250 seconds or greater. Intracoronary nitroglycerin (100-200 μg) was injected through the guide catheter and a 0.014-inch PressureWire X (Abbott) was calibrated and equalized to the guide catheter pressure and advanced to the distal two-thirds of the left anterior descending coronary artery. Coronary function testing was performed with adenosine (Ado), ACh, and Dob in that order. Ado was administered at 140 mcg/kg/min and thermodilution and pressure measurements were made before and 3 minutes after the start of this infusion. Coronary flow reserve (CFR) and index of microvascular resistance (IMR) were measured using commercially available software (CoroFlow, Abbott). CFR was calculated using the ratio of hyperemic blood flow (1/T_mn, hyperemia_) to resting blood flow (1/T_mn, resting_), or simply T_mn, resting_/T_mn, hyperemia_. IMR was calculated using the ratio of hyperemic mean distal coronary artery pressure to hyperemic flow, or simply mean distal coronary artery pressure × T_mn, hyperemia_.^16^ ACh studies were performed 3 minutes after Ado infusion was complete. For this, low doses were given intracoronary to the left main coronary artery at 20 (test dose for safety) then 40 mcg (low dose) over 30 seconds. Resting and hyperemic measurements of T_mn_ and pressure were obtained. Postinfusion FFR was measured and presence or absence of chest pain and electrocardiogram (ECG) changes were recorded. Following this, 100 mcg ACh was administered over 30 seconds to evaluate for epicardial spasm, for which angiography was performed and FFR was repeated. Lastly, Dob was administered peripherally starting at 10 mg/kg/min and increasing in increments of 10 mg/kg/min every 3 minutes until the target heart rate of 85% maximum predicted heart rate was achieved or the maximum rate of infusion of 40 mcg/kg/min was achieved. At this point, resting full-cycle ratio (RFR) and FFR were recorded. Some patients also underwent noninvasive anatomical assessment of the MB with coronary computed tomography angiogram (CTA).

### Interpretation of coronary function testing

HD significant MB was defined as resting RFR^5^, ^17^ ≤0.76 after administration of the final dose of Dob. Coronary endotypes were defined in accordance with COVADIS criteria.^13^ Epicardial spasm was defined as angiographic spasm ≥90% or ischemic FFR ≤0.80 with administration of ACh. The location of the spasm in relation to the bridge segment was recorded. Microvascular spasm was defined as the presence of chest pain and ischemic ECG changes with administration of low-dose ACh in the absence of an ischemic FFR of ≤0.80. Endothelium-independent CMD was defined as CFR <2.0 or IMR ≥25 with administration of Ado. Although not used in the definition of microvascular spasm, ACh CFR and IMR were recorded and considered abnormal if CFR was <1.5 or IMR >31.

### Statistical analyses

Descriptive statistics were performed in the overall cohort. Patients were then divided into 2 groups with RFR ≤0.76 defining HD significant MB and RFR > 0.76 defining HD insignificant MB. Data analysis was performed using Stata version 15 (StataCorp LLC) and R statistical software (The R Foundation). Levene’s test for equality of variances was performed for each variable and the appropriate *t* test was then performed to compare means. Univariate logistic regression was performed to determine CT correlates of bridge significance.

## Results

### Baseline characteristics

In the study, a total of 30 patients were included with a mean age of 47 years and 63% were female (Table 1). Obesity, hypertension, and hyperlipidemia were common. No patients had HFrEF; 6 patients had HFpEF. Medications listed represent those taken prior to cardiac catheterization and study enrollment. All patients had angina, and dyspnea was also common (70%).

**Table 1:** Baseline clinical characteristics.

	All patients (N = 30)
Age, y	47 ± 10
Female	18 (60%)
African-American	4 (19%), n = 21
Hispanic/Latino	4 (17.4%), n = 23
BMI, kg/m^2^	31.3 ± 7.2
>30 kg/m^2^ (obese)	15 (50%)
CAD (nonobstructive)	5 (16.7%)
Hypertension	12 (40%)
HFrEF	0 (0%)
HFpEF	6 (20%)
Diabetes mellitus	2 (6.7%)
Hyperlipidemia	15 (50%)
History of stroke or TIA	1 (3.3%)
COPD/asthma	1 (3.3%)
Obstructive sleep apnea	8 (26.7%)
Angina	30 (100%)
Dyspnea	21 (70%)
Medications	
Beta blocker	10 (33.3%)
ACE inhibitor or ARB	7 (23.3%)
Calcium channel blocker	14 (46.7%)
Aspirin	11 (36.7%)
Statin	13 (43.3%)
Loop diuretic	0 (0%)
P2Y12 inhibitor	1 (3.3%)
Nitrate	2 (6.7%)

Values are mean ± SD or n (%).

ACE, angiotensin-converting enzyme; ARB, angiotensin receptor blocker; BMI, body mass index; CAD, coronary artery disease; COPD, chronic obstructive pulmonary disease; HFpEF, heart failure with preserved ejection fraction; HFrEF, heart failure with reduced ejection fraction; TIA, transient ischemic attack.

### Coronary function testing

**Figure 1:**
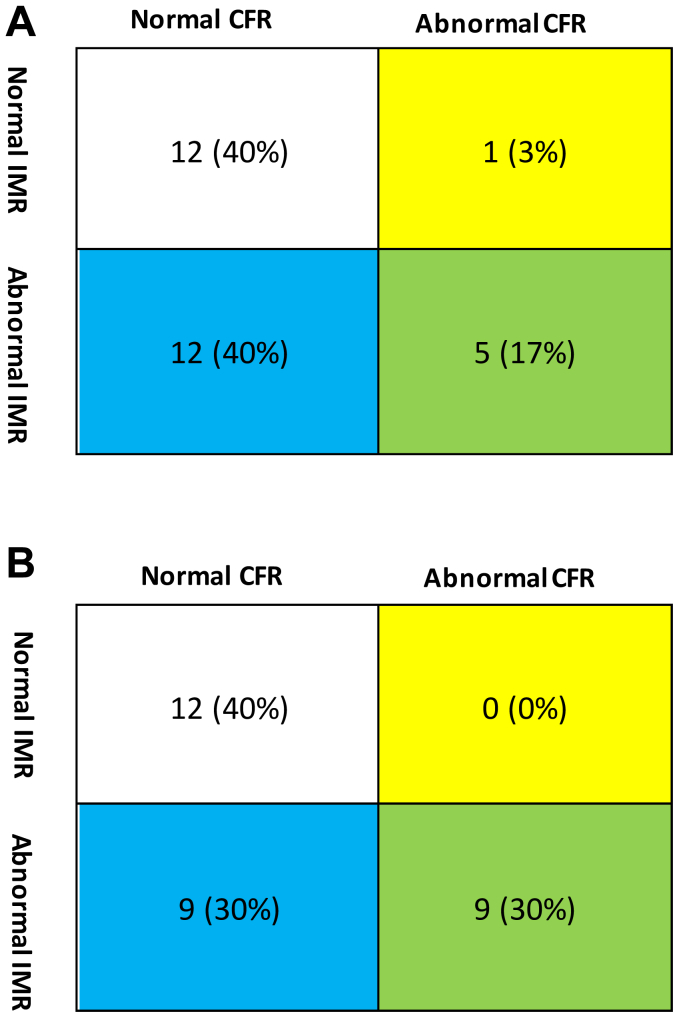
**Adenosine and acetylcholine coronary flow reserve (CFR) and index of microvascular resistance (IMR).** This figure displays the microvascular endotypes of all 30 patients with (**A**) adenosine (Ado) and (**B**) acetylcholine (ACh) testing. For Ado, abnormal CFR was defined as <2.0 and abnormal IMR ≥25. For ACh, abnormal CFR was defined as <1.5 and abnormal IMR >31.

**Figure 2:**
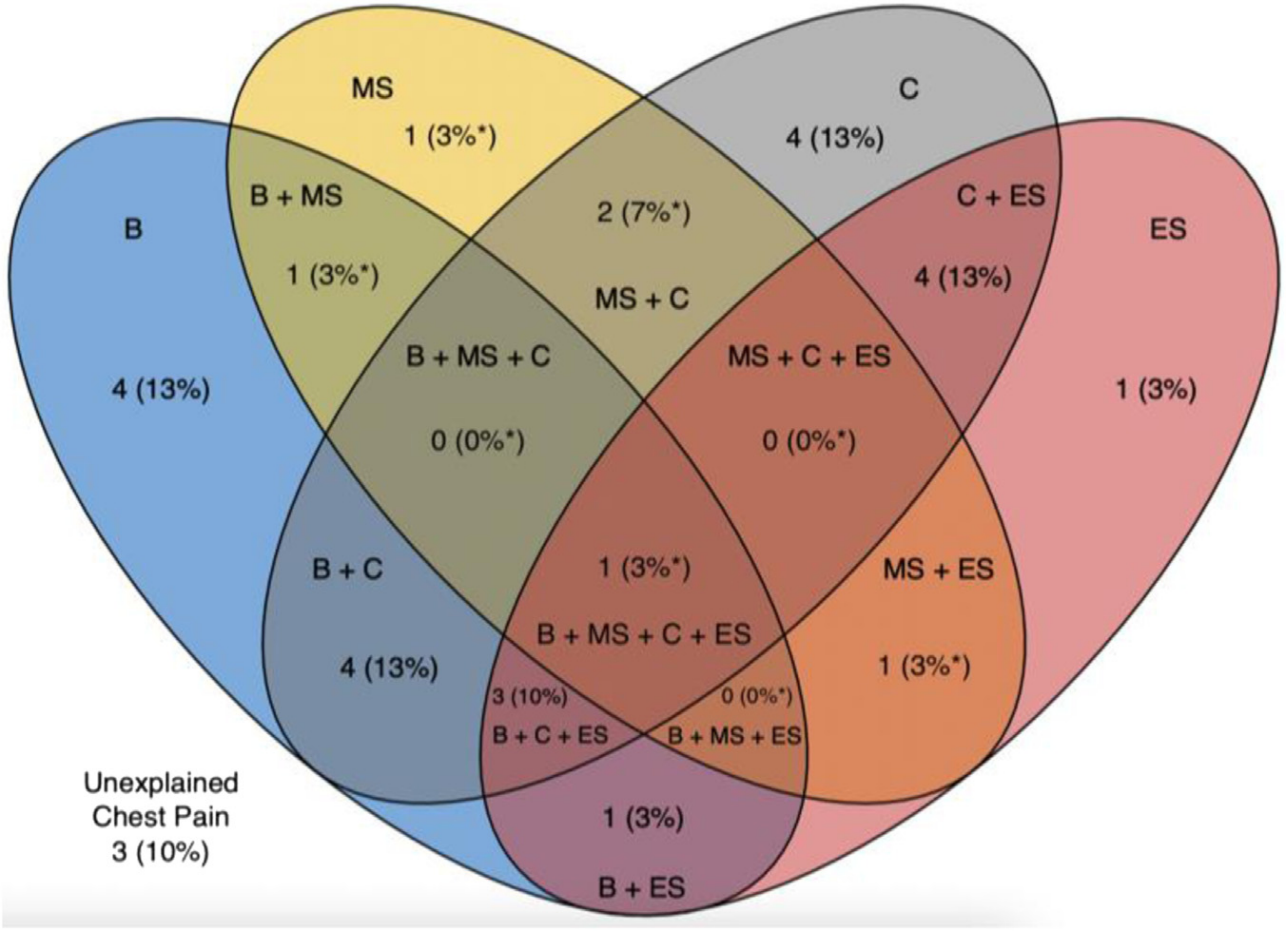
**Coronary endotypes in patients with chest pain and myocardial bridge.** This Venn diagram displays the prevalence of hemodynamically significant myocardial bridge and alternate potential causes of chest pain including epicardial spasm, microvascular spasm, and endothelium-independent coronary microvascular dysfunction (CMD). There is significant overlap between groups with only 3 patients having no potential etiology of chest pain identified. B, hemodynamically significant myocardial bridge; C, endothelium-independent CMD; ES, epicardial spasm; MS, microvascular spasm

Endothelium-independent CMD was common (n = 18, 60.0%) with abnormal IMR more frequent than abnormal CFR (17 [56.7%] vs 6 [20.0%]; Table 2). ACh CFR and IMR were recorded but not used to define microvascular spasms. Abnormal ACh CFR (n = 6, 28.6%) and IMR (n = 12, 57.1%) were common. Of the 18 patients with abnormal ACh CFR, IMR, or both, 7 (38%) had microvascular spasms whereas 3 (25%) of the 12 patients with normal ACh CFR and IMR had microvascular spasms. Rates of abnormal Ado and ACh CFR and IMR are displayed in Figure 1. Epicardial spasm of ≥90% lumen diameter reduction or ischemic FFR occurred in 11 (36.7%) patients and vasoconstriction of 20% to 89% lumen diameter reduction occurred in 9 (30.0%) patients. All patients with ischemic FFR with ACh also had angiographic evidence of epicardial spasm. All spasms occurred within the LAD and were frequently confined to the bridge segment (n = 10, 30.0%) but commonly extended distally (n = 9, 45.0%). One patient had spasms proximal to the bridge segment in addition to within and distal to the bridge. Nine patients had ischemic FFR at low-dose ACh and were therefore excluded from testing for microvascular spasm. Of these 9 patients, 4 did develop chest pain and ECG changes. Of the remaining 21 patients, 6 (28.6%) had microvascular spasm. Only 7 patients had no abnormality (epicardial spasm, microvascular spasm, or endothelium-independent CMD), and 2 of those 7 patients did have mild vasoconstriction (20%-89%) with ACh. These findings are summarized in Figure 2.

**Table 2:** Prevalence of epicardial spasm, microvascular spasm, and endothelium-independent coronary microvascular dysfunction in overall study population.

	All patients (N = 30)
Dob RFR	0.79 ± 0.01
Dob FFR	0.85 ± 0.05 (n = 28)
Adenosine	
Ado CFR	3.3 ± 1.4
Ado IMR	29.8 ± 14.8
Abnormal Ado CFR	6 (20%)
Abnormal Ado IMR	17 (56.7%)
Any endothelium-independent microvascular dysfunction	18 (60%)
Acetylcholine	
Overt epicardial spasm (≥90% or ischemic FFR)	11 (36.7%)
Spasm 20%-89%	9 (30%)
Location of spasm:	
Bridge segment	10 (50%)
Bridge segment + distal	9 (45%)
Bridge segment + distal + proximal	1 (5%)
Microvascular spasm (CP + ECG changes)^a^	6 (28.6%)
ACh CFR^a^	2.4 ± 1.2
ACh IMR^a^	48.8 ± 30.2
Abnormal ACh CFR^a^	6 (28.6%)
Abnormal ACh IMR^a^	12 (57.1%)
Abnormal ACh CFR or IMR^a^	12 (57.1%)
Both endothelium-independent CMD and microvascular spasm^a^	3 (14.3%)
Endothelium-independent CMD, microvascular spasm, or both^a^	14 (66.7%)
No abnormality with Ado and ACh testing	7 (23.3%)

Values are mean ± SD or n (%).

ACh, acetylcholine; Ado, adenosine; CFR, coronary flow reserve; CMD, coronary microvascular dysfunction; CP, chest pain; Dob, dobutamine; ECG, electrocardiogram; FFR, fractional flow reserve; IMR, index of microvascular resistance; RFR, resting full-cycle ratio.

a Excluding obstructive epicardial spasm (ACh FFR ≤ 0.80, n = 9 [30.0%]).

### Hemodynamic significance of the bridge and surgical referral patterns

All patients underwent Dob testing. Fourteen subjects had an HD significant MB on RFR Dob testing whereas 16 subjects had an HD insignificant bridge (Table 3). There was no significant difference in the rates of endothelium-independent CMD or microvascular spasm between those with HD significant versus insignificant MB. There was no significant difference in the rate of epicardial spasm with ACh. Patients referred for surgical intervention had higher rates of HD significant MB and had lower average RFR and FFR. Rates of functional abnormalities were similar between the 2 groups (Table 4).

**Table 3:** Prevalence of epicardial spasm, microvascular spasm, and endothelium-independent coronary microvascular dysfunction by presence of hemodynamically significant myocardial bridge.

	HD significant bridge (RFR ≤ 0.76), n = 14	HD insignificant bridge (RFR > 0.76), n = 16	*P* value
Adenosine			
Ado CFR	2.9 ± 1.3	3.7 ± 1.5	.12
Ado IMR	25.4 ± 9.6	33.8 ± 17.5	.11
Abnormal Ado CFR	4 (28.6%)	2 (12.5%)	.29
Abnormal Ado IMR	7 (50.0%)	10 (62.5%)	.51
Any endothelium-independent microvascular dysfunction	8 (57.1%)	10 (62.5%)	.77
Acetylcholine			
Overt spasm (>90% or ischemic FFR)	5 (35.7%)	6 (37.5%)	.92
FFR low-dose ACh	0.79 ± 0.12	0.79 ± 0.16	.98
Abnormal FFR	4 (28.6%)	5 (31.3%)	.88
Microvascular spasm (CP + ECG changes)^a^	2 (20.0%)	4 (36.4%)	.43
ACh CFR^a^	2.4 ± 1.5	2.4 ± 1.1	.99
ACh IMR^a^	43.7 ± 29.1	53.5 ± 31.7	.47
Abnormal ACh CFR^a^	4 (40.0%)	2 (18.2%)	.29
Abnormal ACh IMR^a^	5 (50%)	7 (63.6%)	.67
Abnormal ACh CFR or IMR^a^	5 (50.0%)	7 (63.6%)	.55

Values are mean ± SD or n (%).

Analyses were performed with the remaining 10 patients in the HD significant myocardial bridge group and 11 patients in the HD insignificant myocardial bridge group.

ACh, acetylcholine; Ado, adenosine; CFR, coronary flow reserve; CP, chest pain; ECG, electrocardiogram; FFR, fractional flow reserve; HD, hemodynamically; IMR, index of microvascular resistance; RFR, resting full-cycle ratio.

a Excluding obstructive epicardial spasm (ACh FFR ≤0.80, n = 9 [30.0%]).

**Table 4:** Prevalence of hemodynamically significant bridge and functional abnormalities by surgical referral status.

	Unroofing surgery, N = 13	No unroofing surgery, N = 17	*P* value
Dob FFR	0.82 ± 0.03 (n = 12)	0.88 ± 0.04 (n = 16)	.001
Dob RFR	0.72 ± 0.08	0.8 ± 0.09	.002
HD significant bridge (RFR <0.76)	10 (76.9%)	4 (23.5%)	.003
Adenosine			
Ado CFR	2.9 ± 1.4	3.7 ± 1.4	.17
Ado IMR	26.8 ± 13.0	32.2 ± 16.0	.33
Abnormal Ado CFR	4 (30.8%)	2 (11.8%)	.21
Abnormal Ado IMR	6 (46.2%)	11 (64.7%)	.33
Any endothelium-independent microvascular dysfunction	7 (53.8%)	11 (64.7%)	.56
Acetylcholine	N = 9	N = 25	
Overt spasm (≥90% or FFR <0.8)	5 (38.5%)	6 (35.3%)	.86
FFR low-dose ACh	0.77 ± 0.11	0.81 ± 0.15	.51
Abnormal FFR	5 (38.4%)	4 (23.5%)	.39
Microvascular spasm (CP + ECG changes)^a^	3 (37.5%)	3 (23.1%)	.50
ACh CFR^a^	2.64 ± 1.1	2.25 ± 1.3	.49
ACh IMR^a^	36.0 ± 17.7	56.7 ± 34.0	.13
Abnormal ACh CFR^a^	2 (25.0%)	4 (30.8%)	.79
Abnormal ACh IMR^a^	4 (50.0%)	8 (61.5%)	.63
Abnormal ACh CFR or IMR^a^	4 (50.0%)	8 (61.5%)	.63

Values are mean ± SD or n (%).

ACh, acetylcholine; Ado, adenosine; CFR, coronary flow reserve; CP, chest pain; Dob, dobutamine; ECG, electrocardiogram; FFR, fractional flow reserve; HD, hemodynamically; IMR, index of microvascular resistance; RFR, resting full-cycle ratio.

a Excluding obstructive epicardial spasm (ACh FFR ≤0.80, n = 11 [32.4%]).

**Figure 3:**
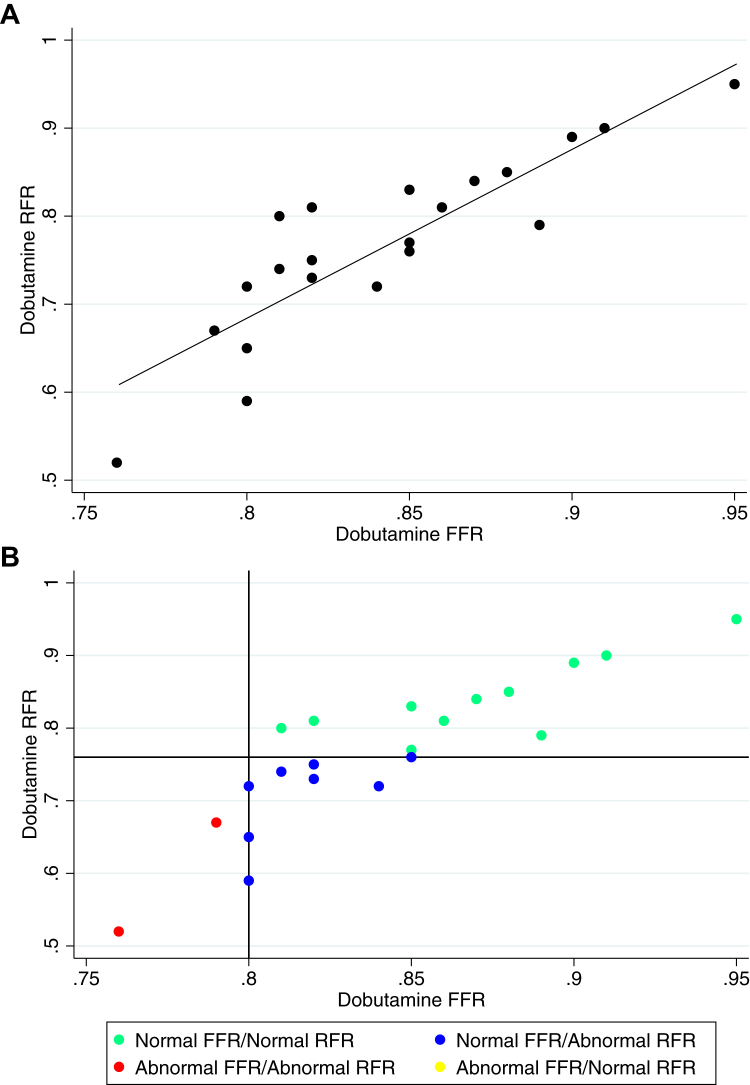
**Correlation of dobutamine resting full-cycle ratio (RFR) and fractional flow reserve (FFR).** Panel (**A**) displays regression of dobutamine RFR vs FFR (R^2^ = 0.81, *P* ≤ .001). Panel (**B**) displays patients divided into 4 groups according to normal/abnormal RFR and FFR. ∗ Indicates 2 patients with the recorded value and † indicates 3 patients with the recorded value. Two patients were excluded from analysis as dobutamine FFR measurements were not recorded.

There was a strong correlation (*R*^2^ = 0.81; *P* < .001) between Dob RFR and FFR (0.79 ± 0.01 vs 0.85 ± 0.05). When divided based on an FFR cutoff of <0.80 to define hemodynamic significance, FFR failed to identify 11 of the 14 patients found to have a significant bridge by RFR assessment (Figure 3). Two patients were excluded from analysis due to lack of FFR measurement.

### Coronary CTA characteristics

Fourteen patients underwent CTA. MB length was significantly longer in patients with HD significant MB than those without. There was no relationship between bridge location (mid vs distal LAD) and hemodynamic significance (Table 5). In univariate analysis, neither CT characteristic was found to predict bridge significance given the 95% CI for the odds ratio crossed 1.

**Table 5:** CT characteristics of the myocardial bridge stratified by hemodynamic significance and logistic regression.

	HD significant bridge (RFR <0.8), n = 6	HD insignificant bridge (RFR ≥0.8), n = 8	*P* value	Odds ratio (95% CI)	*P* value
MB length, mm	27.7 ± 9.3, n = 6	14.2 ± 5.6, n = 6	.012	1.49 (0.89-2.50)	.003
MB location:			.433	3.0 (0.227-39.6)	.383
Mid LAD	5 (83.3%)	5 (62.5%)			
Distal LAD	1 (16.7%)	3 (37.5%)			

Values are mean ± SD or n (%).

HD, hemodynamically; LAD, left anterior descending artery; MB, myocardial bridge; RFR, resting full-cycle ratio.

## Discussion

### Prevalence of CMD and spasm in patients with MB

This prospective study evaluated the prevalence of invasively determined endothelium-independent CMD and microvascular spasm as well as epicardial spasm in patients presenting with angina suspected to be due to MB. We made a number of important observations. Dob RFR reveals positive results in 47%, the majority of which would be missed if using only FFR. Endothelium-independent microvascular dysfunction, microvascular spasm, and epicardial spasm are prevalent in patients with MB. In fact, only 7 of 30 patients had no functional abnormality. Independent of positive RFR, the prevalence of functional disease is high suggesting that multiple abnormalities need to be treated. These findings suggest that chest pain syndromes with MB should be interrogated with both Dob RFR (or RFR equivalent) and coronary function testing (Central Illustration).

**Central Illustration:**
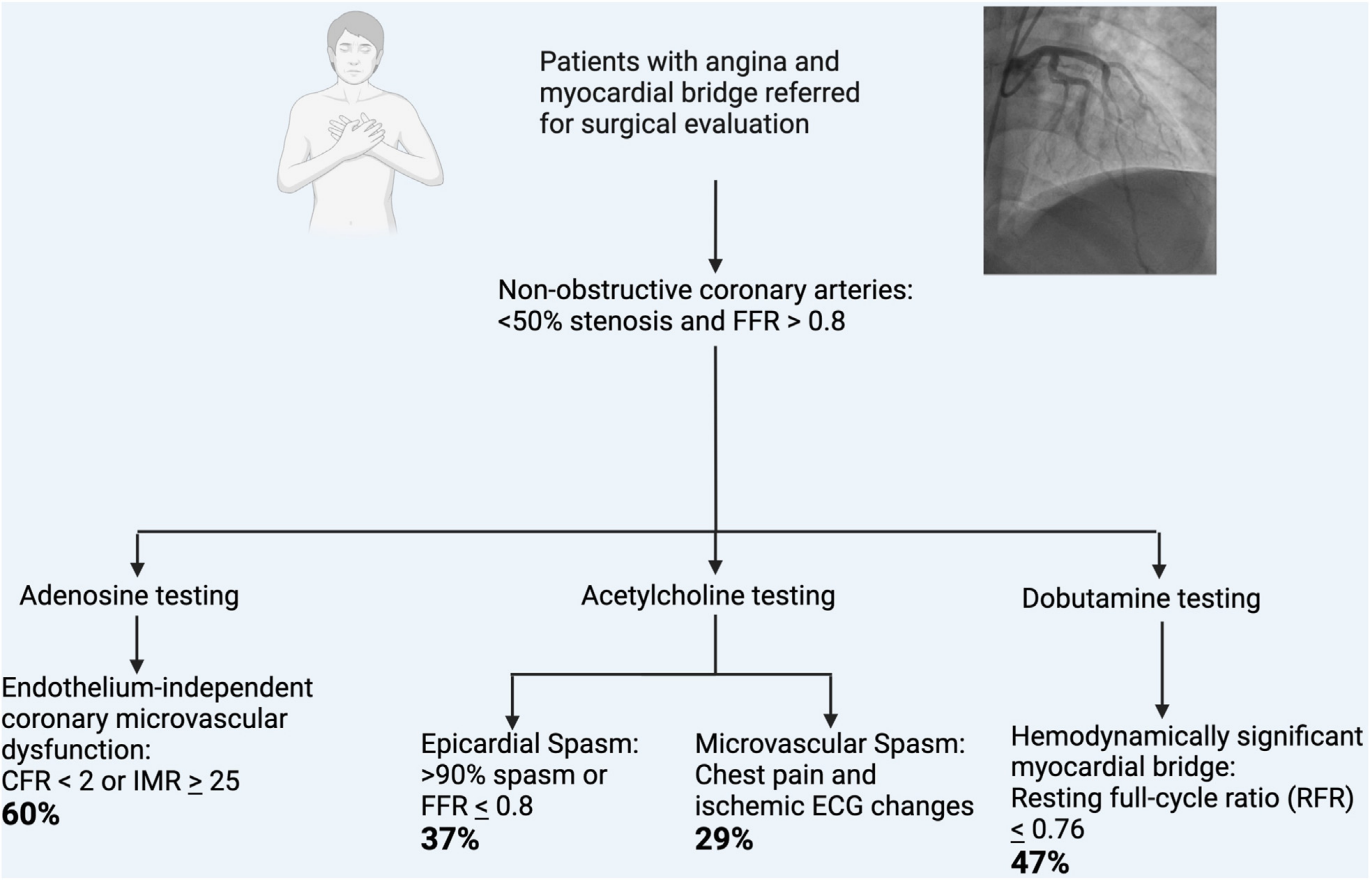
**Evaluation of chest pain in patients with myocardial bridge.** The diagnostic evaluation performed for patients presenting with angina and known myocardial bridge including definitions of hemodynamically significant myocardial bridge, microvascular spasm, epicardial spasm, and endothelium-independent coronary microvascular dysfunction. CFR, coronary flow reserve; FFR, fractional flow reserve; IMR, index of microvascular resistance.

Recent investigations have shown coronary functional abnormalities to be common in patients with angina with estimates ranging from 45%^18^ to 66%.^9^ An early analysis by Lee et al^19^ of patients with angina and no obstructive coronary disease found that among the subset with MB, 60% also had abnormal Ado IMR, abnormal response to ACh, or both. Another study demonstrated that in patients with angina, presence of MB was associated with higher rates of induced coronary vasospasm with ACh testing but was not associated with higher rates of endothelium-independent CMD compared to those patients without MB.^12^ Recent analysis of Lee et al’s cohort found a higher rate of endothelial dysfunction than endothelium-independent CMD in patients with MB, although their definition used a narrower definition of CMD which included only IMR.^20^ When Doppler flow velocity was measured, another group found that MB was associated with high rates of both epicardial and microvascular endothelial dysfunction.^8^ Taken together, these studies confirm the presence of microvascular spasm, endothelium-independent CMD, and epicardial vasospasm in symptomatic patients who happen to have MB. Our study contributes to the body of literature that these functional abnormalities are common potential causes of angina and ischemia with nonobstructive coronary arteries, even in patients with known MB being evaluated for surgical therapy.

We evaluated exclusively patients whose chest pain syndrome was thought to be due to MB, yet 53% of these patients did not have HD significant MB. Endothelium-independent CMD, microvascular spasm, and epicardial spasm could explain, at least in part, their chest pain syndrome. Additionally, this study provides a comprehensive invasive evaluation of CMD with both Ado and ACh allowing for delineation between endothelium-independent microvascular dysfunction and microvascular spasm. Further, there was no difference in rates of functional abnormalities based on the hemodynamic significance of MB. Our study provides important new insight by including an assessment of microvascular spasm with a recording of chest pain and ECG changes with ACh infusion as well as endothelium-independent CMD with Ado testing modeled after definitions based on current expert consensus.^12^^,^^21^

Epicardial spasm with ACh infusion was common supporting prior studies that have shown increased rates of spasm in patients with MB.^10^^,^^22^ A study by Yoshino et al^23^ demonstrated that patients with impaired coronary blood flow with ACh challenge had significantly decreased FFR across the bridge segment with Dob infusion which was not observed in those patients with normal increase in coronary blood flow with ACh. This finding supports the idea that ischemia across an MB can be worsened by coexistent spasm. In those with epicardial spasms, the majority had spasms localized to the bridge segment though a significant number had spasms of the segment distal to the bridge as well. No patients had spasms noted in the left circumflex. This supports the previously described localization of spasm within the bridge segment. Multiple mechanisms have been proposed to explain this phenomenon including turbulent flow and increased wall shear stress in the bridge segment leading to endothelial damage^10^^,^^22^ as well as lowered nitric oxide production in the bridge segment.^24^ The clinical importance of assessment for epicardial spasm in patients with MB is highlighted by data demonstrating that those patients with MB and vasospasm have higher rates of recurrent angina than those with MB alone.^25^ Microvascular spasm was also common and not affected by the hemodynamic significance of the MB. Additionally, objective measures of coronary blood flow (CFR and IMR) during ACh infusion do not suggest an increase in microvascular resistance in those with HD significant MB.

Our results demonstrate the importance of evaluating for functional abnormalities in patients with MB before concluding that compression of the bridge is the culprit for the patient’s angina. Further, both Ado and ACh studies are necessary to comprehensively assess for alternative etiologies of chest pain in patients with known MB.

### Hemodynamic significance of the MB and surgical referral patterns

This study sought to determine the rate of HD significant MB and whether hemodynamic significance impacted the prevalence of functional abnormalities with Ado and ACh testing. Epicardial spasm, microvascular spasm, and endothelium-independent CMD were prevalent and rates were not affected by the hemodynamic significance of the bridge.

A significant number of patients in our study, all of whom presented with chest pain, had HD insignificant MB with Dob testing. This highlights the importance of functional coronary assessment in patients with known bridges as a significant number of patients will have an HD insignificant bridge unlikely to explain their symptoms. Additionally, the presence of an MB may increase the risk of epicardial and microvascular spasm as well as endothelium-independent CMD regardless of hemodynamic significance making comprehensive coronary functional assessment all the more important. There was a limited correlation between CT bridge characteristics and hemodynamic significance. Given the small number of patients with complete CT data, this analysis is primarily exploratory, and no conclusions can be drawn on the potential role of CT in predicting the hemodynamic significance of the MB based on these findings.

Existing studies of CMD and epicardial spasm in patients with MB have not stratified by the hemodynamic significance of the bridge. If medical therapy fails, surgical unroofing has demonstrated symptomatic benefit though a significant number of patients continue to experience angina.^26^^,^^27^ In our patient cohort, surgical referral patterns were primarily based on the hemodynamic significance of the bridge. Given the prevalence of abnormalities during functional testing in our cohort, those who do not benefit from unroofing surgery may have other underlying causes of angina. Identification of coexisting epicardial or microvascular spasm and endothelium-independent CMD in patients with MB is important prior to considering surgical intervention to ensure patients are selected appropriately for surgery. As it is not known whether surgical unroofing will improve the functional abnormalities associated with the bridge segment, knowledge of coronary endotypes can assist in appropriate selection of surgical candidates and treatment should angina recur postoperatively.

### Pharmacologic agents and definition of hemodynamic significance

Although previous studies have shown near identical ischemic cutoffs for FFR and diastolic FFR (d-FFR) under resting conditions,^28^ overshooting of the systolic pressure due to compression of the bridge segment can falsely raise the FFR in hyperemic conditions. Diastolic measurements such as d-FFR or instantaneous wave-free ratio (iFR) avoid measurement errors due to systolic bridge compression.^5^ As the RFR scans the full cardiac cycle for the point of lowest Pd to Pa ratio, it is a reasonable alternative to the iFR. The VALIDATE-RFR trial demonstrated diagnostic equivalence between iFR and RFR.^17^ Because the only commercially available wire (PressureWire X) to measure CFR and IMR measures RFR but not iFR, we used RFR as our index measurement for detection of hemodynamic significance of the MB to avoid an additional wire exchange. Studies such as FAME 2^29^ and DEFER^30^ have proposed ischemic FFR cutoffs of ≤0.80 and <0.75, respectively. At our center, we use an RFR cutoff of 0.76 to define hemodynamic significance in line with previously established d-FFR ischemic cutoff.^5^ For evaluation for ischemia with acetylcholine or adenosine, we used an FFR ischemic cutoff of 0.80 in accordance with FAME 2.^29^ FFR was not used to determine hemodynamic significance of the bridge. In our study, RFR assessment increased the detection rate of HD significant bridges over standard FFR measurements.

### Coronary physiology study technique

Although a strict coronary physiology protocol was followed in the catheterization laboratory at our center to ensure internal consistency, we recognize that techniques are not fully standardized across centers. In our protocol, we avoid wire exchange between Ado and ACh testing which results in assessment of epicardial and microvascular spasm with a wire in place. Previous studies have employed this technique without an increase in the rate of epicardial spasm.^31^ We selected a stringent definition of abnormal Ado CFR (<2) to increase specificity. Common cutoffs range from 2 to 2.5 for Ado CFR, and CFR <2 on both invasive and noninvasive assessment has been associated with a higher risk of cardiovascular events and death.^32^ Similar to other centers, our radial cocktail includes nitroglycerin. Given the short duration of action of nitroglycerin, this inclusion should not alter the ACh results. We also recognize there is variability in protocols across centers including with duration of Ado infusion. Our methods are consistent with other centers and have been employed by our group in prior studies; however, standardization of practice patterns across centers would aid in ensuring results are replicable.

### Study limitations

This was a single-center, single-operator study with a small sample size all of which may limit generalizability. Additionally, coronary physiology study techniques vary across centers which decreases standardization and could, in theory, affect results.

## Conclusion

This study demonstrates that epicardial spasm, microvascular spasm, and endothelium-independent CMD are all common in patients presenting with chest pain previously attributed to an MB. The prevalence of functional disease remains high irrespective of positive Dob RFR. RFR was more sensitive than FFR in detecting HD significant MB. These findings suggest that chest pain syndromes with MB should be interrogated with functional testing as outlined in this study.

## References

[1] Hostiuc S., Negoi I., Rusu M.C., Hostiuc M. (2018). Myocardial bridging: a meta-analysis of prevalence. J Forensic Sci.

[2] Lee M.S., Chen C.H. (2015). Myocardial bridging: an up-to-date review. J Invasive Cardiol.

[3] Medina F., Estrada A., Fernandez C. (2023). Use of intravascular ultrasound and coronary angiography to measure the prevalence of myocardial bridge in heart transplant patients. Am J Cardiol.

[4] Tang K., Wang L., Shi R. (2011). The role of myocardial perfusion imaging in evaluating patients with myocardial bridging. J Nucl Cardiol.

[5] Escaned J., Cortés J., Flores A. (2003). Importance of diastolic fractional flow reserve and dobutamine challenge in physiologic assessment of myocardial bridging. J Am Coll Cardiol.

[6] Aleksandric S.B., Djordjevic-Dikic A.D., Dobric M.R. (2021). Functional assessment of myocardial bridging with conventional and diastolic fractional flow reserve: vasodilator versus inotropic provocation. J Am Heart Assoc.

[7] Tarantini G., Barioli A., Nai Fovino L. (2018). Unmasking myocardial bridge-related ischemia by intracoronary functional evaluation. Circ Cardiovasc Interv.

[8] Bairey Merz CN., Shaw L.J., Reis S.E. (2006). Insights from the NHLBI-Sponsored Women’s Ischemia Syndrome Evaluation (WISE) Study: Part II: gender differences in presentation, diagnosis, and outcome with regard to gender-based pathophysiology of atherosclerosis and macrovascular and microvascular coronary disease. J Am Coll Cardiol.

[9] Sara J.D., Widmer R.J., Matsuzawa Y., Lennon R.J., Lerman L.O., Lerman A. (2015). Prevalence of coronary microvascular dysfunction among patients with chest pain and nonobstructive coronary artery disease. JACC Cardiovasc Interv.

[10] Sara J.D.S., Corban M.T., Prasad M. (2020). Prevalence of myocardial bridging associated with coronary endothelial dysfunction in patients with chest pain and non-obstructive coronary artery disease. EuroIntervention.

[11] Teragawa H., Fukuda Y., Matsuda K. (2003). Myocardial bridging increases the risk of coronary spasm. Clin Cardiol.

[12] Teragawa H., Oshita C., Uchimura Y. (2022). The impact of myocardial bridging on the coronary functional test in patients with ischaemia with non-obstructive coronary artery disease. Life (Basel).

[13] Kunadian V., Chieffo A., Camici P.G. (2020). An EAPCI expert consensus document on ischaemia with non-obstructive coronary arteries in collaboration with European Society of Cardiology working group on coronary pathophysiology & microcirculation endorsed by Coronary Vasomotor Disorders International Study Group. Eur Heart J.

[14] Mirzai S., Patel B., Balkhy H.H. (2019). Robotic totally endoscopic off-pump unroofing of left anterior descending coronary artery myocardial bridge: a report of two cases. J Card Surg.

[15] Naidu SS, Abbott JD, Bagai J (2021). SCAI expert consensus update on best practices in the cardiac catheterization laboratory. Catheter Cardiovasc Interv.

[16] Dryer K., Gajjar M., Narang N. (2018). Coronary microvascular dysfunction in patients with heart failure with preserved ejection fraction. Am J Physiol Heart Circ Physiol.

[17] Svanerud J., Ahn J.-M., van Jeremias A. (2018). Validation of a novel non-hyperaemic index of coronary artery stenosis severity: the resting full-cycle ratio (VALIDATE RFR) study. EuroIntervention.

[18] Lee S.H., Shin D., Lee J.M. (2022). Clinical relevance of ischemia with nonobstructive coronary arteries according to coronary microvascular dysfunction. J Am Heart Assoc.

[19] Lee B.-K., Lim H.-S., Fearon W.F. (2015). Invasive evaluation of patients with angina in the absence of obstructive coronary artery disease. Circulation.

[20] Pargaonkar V.S., Kimura T., Kameda R. (2021). Invasive assessment of myocardial bridging in patients with angina and no obstructive coronary artery disease. EuroIntervention.

[21] Samuels B.A., Shah S.M., Widmer R.J. (2023). Comprehensive management of ANOCA, part 1—definition, patient population, and diagnosis: JACC state-of-the-art review. J Am Coll Cardiol.

[22] Kim J.W., Park C.G., Suh S.Y. (2007). Comparison of frequency of coronary spasm in Korean patients with versus without myocardial bridging. Am J Cardiol.

[23] Yoshino S., Cassar A., Matsuo Y. (2014). Fractional flow reserve with dobutamine challenge and coronary microvascular endothelial dysfunction in symptomatic myocardial bridging. Circ J.

[24] Masuda T., Ishikawa Y., Akasaka Y., Itoh K., Kiguchi H., Ishii T. (2001). The effect of myocardial bridging of the coronary artery on vasoactive agents and atherosclerosis localization. J Pathol.

[25] Nam P., Choi B.G., Choi S.Y. (2018). The impact of myocardial bridge on coronary artery spasm and long-term clinical outcomes in patients without significant atherosclerotic stenosis. Atherosclerosis.

[26] Hemmati P., Schaff H.V., Dearani J.A., Daly R.C., Lahr B.D., Lerman A. (2020). Clinical outcomes of surgical unroofing of myocardial bridging in symptomatic patients. Ann Thorac Surg.

[27] Boyd J.H., Pargaonkar V.S., Scoville D.H. (2017). Surgical unroofing of hemodynamically significant left anterior descending myocardial bridges. Ann Thorac Surg.

[28] Abe M., Tomiyama H., Yoshida H., Doba N. (2000). Diastolic fractional flow reserve to assess the functional severity of moderate coronary artery stenoses: comparison with fractional flow reserve and coronary flow velocity reserve. Circulation.

[29] De Bruyne B., Pijls N.H.J., Kalesan B. (2012). Fractional flow reserve-guided PCI versus medical therapy in stable coronary disease. N Engl J Med.

[30] Pijls N.H.J., van Schaardenburgh P., Manoharan G. (2007). Percutaneous coronary intervention of functionally nonsignificant stenosis: 5-year follow-up of the DEFER Study. J Am Coll Cardiol.

[31] Nardone M., McCarthy M., Ardern C.I. (2021). Characterization of the human coronary microvascular response to multiple hyperaemic agents. CJC Open.

[32] Taqueti V.R., Shaw L.J., Cook N.R. (2017). Excess cardiovascular risk in women relative to men referred for coronary angiography is associated with severely impaired coronary flow reserve, not obstructive disease. Circulation.

